# Effects of COVID-19 pandemic on psychiatric and psychological consultation-liaison contacts in a general hospital in North-East of Italy: a retrospective study

**DOI:** 10.3389/fpsyt.2024.1414248

**Published:** 2024-11-06

**Authors:** Eleonora Prina, Alice Marquis, Federico Tedeschi, Laura Rabbi, Damiano Salazzari, Mario Ballarin, Marianna Purgato, Giovanni Ostuzzi, Valeria Donisi, Cinzia Perlini, Michela Rimondini, Lidia Del Piccolo, Francesco Amaddeo

**Affiliations:** ^1^ Section of Psychiatry, Department of Neurosciences, Biomedicine and Movement Sciences, University of Verona, Verona, Italy; ^2^ Section of Clinical Psychology, Department of Neurosciences, Biomedicine and Movement Sciences, University of Verona, Verona, Italy

**Keywords:** consultation-liaison, psychiatry, psychology, COVID-19 pandemic, mental health services, general hospitals

## Abstract

**Background:**

The COVID-19 pandemic has prompted significant changes in healthcare, particularly affecting psychiatric and psychological Consultation-Liaison (CL) services in general hospital settings.

**Aim:**

To assess the effects of COVID-19-related restrictions on utilization of psychiatric and psychological CL services in Northeast Italy during 2020, and to compare it to the use of services in the previous year (2019).

**Methods:**

The study collected data on psychiatric and psychological consultations in 2019 and 2020 from a hospital database. It categorizes consultations by type of patient (inpatient or outpatient) and referral source (hospital wards, general practitioners, other specialists). Pandemic-related restrictions were classified as “lockdown,” “intermediate restrictions,” and “no or reduced restrictions” based on the Covid Stringency Index (CSI). Poisson regression models were employed to analyze the data.

**Results:**

The findings reveal a significant 28% increase in the number of psychiatric and psychological consultations in 2020. Consultations for outpatients increased by 51%, while those for inpatients decreased by 11%. However, the lockdown and intermediate restriction phases were deemed responsible of a decrease of 42.9% and 19.5% in consultations respectively.

**Discussion:**

This study highlights the persistent psychological burden during the COVID-19 pandemic, alongside reduced CL services due to lockdown measures. Integrating telemedicine into these types of services becomes imperative for meeting patient needs during restrictions. These findings can inform policies and practices to improve effective mental health care delivery during and beyond pandemics. Future research should explore the impact of pandemic-related restrictions on mental healthcare across settings and clinical factors affecting service accessibility.

## 1 Introduction

Psychiatric and psychological Consultation-Liaison (CL) services are specialized in providing psychiatric and psychological care in collaboration with various healthcare practitioners, typically within general hospital settings (1–3). These professionals play an active role in addressing psychiatric symptoms, including atypical clinical presentations, diagnosing complex cases (4, 5), dealing with stress management and other areas of maladaptive health reactions and behaviors and helping patients in enhancing their coping strategies (6).

The COVID-19 pandemic has required significant changes in the daily activity of psychiatric and psychological CL services to maintain a high standard of clinical care (7–9). Specifically, clinical challenges for psychiatric and psychological services in community settings included mitigating the psychological effects of the pandemic on patients, responding to the immediate needs of individuals with severe mental illnesses, recognizing and treating neuropsychiatric symptoms associated with COVID-19, and providing support to healthcare professionals (5, 10–13). For patients hospitalized with COVID-19 infection, researchers indeed observed symptoms of anxiety, stress, depression, increased suicidal behavior, alcohol consumption, and psychotic symptoms (5, 14–16).

In addition to the above challenges, an essential aspect of CL psychiatry and psychology involves managing neuropsychiatric manifestations and identifying symptoms not typically associated with psychiatric disorders, both during the acute and post-acute pandemic phase. In the acute phase, COVID-19 patients appear to be at risk of a range of neuropsychiatric symptoms and disorders, including delirium, encephalopathy, impaired consciousness, depressed mood, anxiety, and insomnia (17–19), with fatigue, cognitive deficits, depression, and post-traumatic symptoms in the post-acute phase (17, 20–23). Overall, such clinical manifestations deeply impact on survivors’ physical and mental quality of life (20).

Following the first documented cases on February 21, 2020, Italy experienced significant impacts from the COVID-19 pandemic, ranking second only to China in terms of affected countries (24). Consequently, on March 9, the government implemented stringent measures to restrict population movement and prevent gatherings. This was followed by a national quarantine declaration on March 11, which mandated the closure of all non-essential businesses and restricted movement except for essential reasons such as work activities and health issues, aimed at reducing the spread of the coronavirus. Subsequently, on April 26, the Italian government announced the conclusion of the lockdown, leading to a gradual resumption of economic activities starting from May 4 (24). However, due to an increase of COVID-19 cases in October 2020, more stringent restrictions were imposed, culminating in the establishment of a regional-based system on November 3, which implemented varying levels of measures based on the pandemic’s trend in different country’s areas (25).

Despite this challenging context, the Italian literature highlights a substantial decrease in both the total number of psychiatric emergency consultations and psychiatric admission rates during the lockdown period (26, 27). However, there is a lack of evidence regarding the organizational changes in psychiatric and psychological CL services in Italy and especially their long-term consequences on patients’ mental health during the COVID-19 pandemic (28, 29).

The present study aims to assess the effects of lockdown and intermediate restrictions on the clinical activities of a psychiatric and psychological CL service in a General Hospital in Northeast Italy according to the characteristics of referrals and through a comparison of similar time-periods in 2019 and 2020.

## 2 Materials and methods

This is a retrospective study conducted on all psychiatric and/or psychological consultations of adult patients (>18 years of age, inpatient and/or outpatient) in the years 2019 and 2020. This project complied with the principles of the Declaration of Helsinki with respect to medical research involving human subjects. Approval for the study was obtained from the local Ethics Committee (Prog. 3327CESC).

### 2.1 Setting

This study was conducted at the Verona University Hospital Trust, situated in the northeast of Italy. The Verona University Hospital is the second largest hospital trust in Italy in 230 terms of the number of beds (1617 beds in 2019) and the fifth largest in terms of admissions (44,593 planned and urgent admissions, and 12,214 day-hospital admissions in 2019). Outpatient contacts were about 3.7 millions in 2019. The Verona Trust treats patients coming from all over Italy (17% come from outside the Veneto Region). The trust is formed by two hospitals, located in two different parts of the city, both having psychiatric and psychological services. The consultations considered in this paper were carried out by the “Psychosomatic and Psychological Medicine Unit” and the “Clinical Psychology Unit” of one of the two hospitals, the G.B. Rossi Hospital, which has a capacity of about 500 beds for inpatients and dedicated clinics for day-hospital and day surgery care (delivering about one-third of all admissions and outpatient contacts produced by the trust).

The two Units receive requests for consultation through an online platform from all G.B. Rossi Hospital services, including inpatients, day-patients and outpatients undergoing treatment for organic diseases. They are available from Monday to Friday. Consultations for inpatients are usually conducted within 48 hours after receiving the request, while consultations for day-patients and outpatients are scheduled within few weeks (mainly 2 weeks). Along with in-person or telephone consultations with the requesting medical and/or psychological staff, written reports and diagnoses are provided. The clinical team is composed of psychiatrists, residents in psychiatry, psychologists, psychologists in training and psychotherapists.

For urgent requests from the Emergency Room or other hospital wards, a pool of psychiatrists from another unit (Psychiatry Unit) is available on-call. Furthermore, the “Psychosomatic and Psychological Medicine Unit” and the “Clinical Psychology Unit” serve as a center for the diagnosis and treatment of psychosomatic disorders with several dedicated ambulatories (i.e.: for patients with multiple sclerosis, mastocytosis, functional neurological disorders, hematological diseases, etc.). Additionally, they provide as psychological and psychiatric support to health care professionals. Patients seeking consultation can be also referred by their general practitioners (GPs) or other specialists outside the Verona hospital.

### 2.2 Data source

All data on patients and contacts were obtained from the Hospital Clinical Database (HCD): this database collects all medical records, including planned consultation requests from both surgical and medical wards units. These requests come from the hospital, and also from GPs and other specialists outside the hospital. The database includes comprehensive information regarding the patient clinical history, and number and characteristics of consultations.

During the pandemic, some hospital wards have been completely dedicated to COVID-19 patients, the requests from these wards were classified as “COVID-19-related” consultations.

Information about restrictions and lockdown adopted in Italy by the Government has been obtained using the Covid Stringency Index (CSI). The CSI (25) is a composite measure that assesses the strictness of various COVID-19 response indicators, by considering factors such as school closures, workplace closures, and travel bans. The index has a score ranging from 0 to 100, with higher values indicating more stringent measures implemented in response to the pandemic. We applied the CSI related to Italy.

### 2.3 Data analysis

#### 2.3.1 Comparison between year 2019 and year 2020

We calculated the total number of contacts in the two-year period 2019-2020. The analyses have been conducted considering the type of patients (inpatient and outpatient), in combination with sociodemographic variables, and then with type of referral (hospital wards and external referrals). In this latter case, consultations, both for outpatient and for inpatients, were categorized into three main groups: (1) Outpatients from General Hospital (divided into psychiatric or psychological consultations), (2) Inpatients from General Hospital (divided into surgical or medical wards), (3) Outpatients referred to the CL service by GPs and other specialists outside the hospital (divided again into psychiatric or psychological consultations). Patients who are admitted to the hospital frequently have interaction with both psychiatrists and psychologists during their stay, for this reason and in order to minimize the number of analyses carried out, inpatient consultations were solely categorized based on referral wards. Sociodemographic variables were citizenship (Italian vs. Other); age categories (18-24, 25-44, 45-64, and 65+), and gender (male or female).

To test the equality of the number of contacts between 2019 and 2020 for each variable and globally, the conditional exact binomial test on the equality of proportions has been used. This test, proposed by Przyborowski and Wilenski (30), is suitable for analyzing Poisson variables.pt?>

#### 2.3.2 Identification of the impact of restrictive measures on the number of clinical contacts

Our dataset was composed of the weeks of 2019 and 2020, and the CSI has been adopted as the primary variable of interest to measure the extent of pandemic-related restrictions. The CSI, measured daily for Italy, served to categorize the weeks of 2020 (and 2019 for comparative analysis) into three distinct periods: “no or reduced restrictions,” (average CSI below 0.7) “intermediate restrictions,” (average CSI between 0.7 and 0.8) and “lockdown” (average CSI above 0.8).

To address the discrete nature of the outcome variable, which is the number of contacts per working day, a Poisson regression model has been employed. Robust standard errors were incorporated to account for overdispersion.

Given the variability in the number of working days among weeks due to public holidays and the extended last week of each year, the variable representing the number of working days per week was included. This adjustment allows to normalize contact numbers by considering variations in working days.

Considering that COVID-19 restrictions encompassed travel limitations, an indicator variable has been introduced to accommodate weeks that featured public holidays during which inter-regional travel was either prohibited or discouraged. This control variable ensures that potential reductions in contacts during holiday weeks are appropriately considered.

In summary, this methodology allows us to examine the impact of restriction levels on contacts number, while considering the discrete nature of the outcome and accounting for factors such as the number of working days and travel restrictions.

The study employed a “difference-in-differences” approach, as outlined by Higgins et al. (31). Specifically, this approach involved comparing the difference in the number of contacts between the years 2019 and 2020 during weeks characterized by lockdown and intermediate restrictions with the corresponding difference in weeks presenting no or reduced restrictions. This comparison allowed us to interpret the effects of the pandemic-related restrictions. To implement this approach, three indicators have been incorporated into the regression analysis: one related to the year 2020 and two related to specific periods within that year (one for weeks with intermediate restrictions and another for weeks with lockdown measures).

Our parameters of interest were the two interactions between the year indicator and those related to the level of restrictions in 2020. We conducted both a comprehensive regression analysis considering all contacts and separate regressions for each type of contact, by distinguishing between outpatient and admission contacts.

Furthermore, to assess whether variations in the effects of restrictions across different service types were statistically significant, a global test has been performed using a Poisson regression model. This model incorporated an exposure variable representing the number of working days in each week, allowing to account for potential differences in contact patterns arising from variations in working days.

## 3 Results

In the year 2020, a 28% increase in the number of psychiatric and psychological consultations was observed when compared to 2019 (**Table 1**). In particular, consultations requested for outpatients increased by 51%, and those requested for inpatients decreased by 11%. With respect to sociodemographic variables, all categories showed a similar trend towards an increase of the overall number of contacts and outpatient contacts, while overall inpatient contacts decreased, with the exception of young (18-24 years old), elderly (> 65 years old), and female.

**Table 1:** Number of consultations requested during years 2019 and 2020 by patients’ sociodemographic characteristics.

	Outpatients				Inpatients				All patients			
	2019	2020	Δ 2019-20 (%)	P *	2019	2020	Δ 2019-20 (%)	P *	2019	2020	Δ 2019-20 (%)	P *
Citizenship												
** Italian**	1,046	1,561	+515 (+49.2%)	**<0.001**	500	435	-65 (-13.0%)	**0.036**	1,546	1,996	+450 (+29.1%)	**<0.001**
** Other**	42	78	+36 (+85.7%)	**0.001**	56	33	-23 (-41.1%)	**0.019**	98	111	+13 (+13.3%)	0.407
Age												
** 18-24**	105	136	+31 (+29.5%)	0.053	17	21	+4 (+23.5%)	0.627	122	157	+35 (+28.7%)	**0.042**
** 25-44**	325	637	+312 (+96.0%)	**<0.001**	92	46	-46 (-50.0%)	**<0.001**	417	683	+266 (+63.8%)	**<0.001**
** 45-64**	496	566	+70 (+14.1%)	0.034	225	186	-39 (-17.3%)	0.061	721	752	+31 (+4.3%)	0.434
** 65+**	162	282	+120 (+74.1%)	**<0.001**	282	289	+7 (+2.5%)	0.802	444	571	+127 (+28.6%)	**<0.001**
Gender												
** Female**	727	1,161	+434 (+59.7%)	**<0.001**	294	296	+2 (+0.7%)	0.967	1,021	1,457	+436 (+42.7%)	**<0.001**
** Male**	367	486	+119 (+32.4%)	**<0.001**	326	253	-73 (-22.4%)	**0.003**	693	739	+46 (+6.6%)	0.214
**Total**	1,094	1,647	+553 (+50.5%)	**<0.001**	621	551	-70 (-11.3%)	**0.044**	1,715	2,198	+483 (+28.2%)	**<0.001**

^*^ P-value of conditional exact binomial test on equality of proportions. Significant results marked in bold.

In terms of type of consultations (**Table 2**), the comparison between 2019 and 2020 showed an increase in psychological consultations for outpatients, both requested by GPs or other specialists outside the hospital (+60.4%), and by specialists working in hospital wards (+50.6%). Also, the number of both types of psychiatric consultations increased in 2020, respectively of 35.7% and 38.7%. The number of consultations for inpatients decreased in 2020 by about 11%, such decrease being statistically significant both globally and for requests from surgical wards (-33.5%).

**Table 2:** Number of consultations requested during years 2019 and 2020 by type of consultation.

Consultation types	2019	2020	Δ 2019-20 (%)	P ^±^
**All consultations for outpatients**	1,094	1,647	+553 (+50.5%)	**<0.001**
Psychological consultations from GPs or other specialists	444	712	+268 (+60.4%)	**<0.001**
Psychological consultations from hospital wards	326	491	+165 (+50.6%)	**<0.001**
Psychiatric consultations from GPs or other specialists	182	247	+65 (+35.7%)	**0.002**
Psychiatric consultations from hospital wards	142	197	+55 (+38.7%)	**0.003**
**All consultations for inpatients**	621	551	-70 (-11.3%)	**0.044**
Consultations from surgical wards	179	119	-60 (-33.5%)	**0.001**
Consultations from medical wards	442	432	-10 (-2.3%)	0.761
**Total**	1,715	2,198	+483 (+28.2%)	**<0.001**

**^±^** P-value of conditional exact binomial test on equality of proportions. Significant results marked in bold.

In units that request more psychiatric and psychological consultations (**Table 3**) (more than 130 in the two years), the increase was significant for outpatients in care at the neurology unit (+91.0%), infectious diseases unit (+56.1%), and pain therapy unit (+100.0%). Significant decreases were observed in requests from Pancreas and General Surgery for inpatients (-30.5%), and from Maxillofacial surgery for outpatients (-71.7%).

**Table 3:** Number of consultations requested during years 2019 and 2020 by referrals.

Referrals ^*^	2019	2020	Δ 2019-20 (%)	*P* ^±^
**Neurology outpatient**	134	256	+122 (+91.0%)	**<0.001**
**Infectious diseases outpatient**	98	153	+55 (+56.1%)	**0.001**
**Internal medicine inpatient**	75	80	+5 (+6.7%)	0.748
**Neurology inpatient**	63	79	+16 (+25.4%)	0.208
**Pain therapy outpatient**	47	94	+47 (+100.0%)	**<0.001**
**Pancreas and General Surgery inpatient**	82	57	-25 (-30.5%)	**0.041**
**Maxillofacial surgery outpatient**	106	30	-76 (-71.7%)	**<0.001**

^*^ Only hospital wards recording more than 130 contacts in the 2-year period are showed in the table.

**^±^** P-value of conditional exact binomial test on equality of proportions. Significant results marked in bold.

Poisson regression analyses (**Table 4**) revealed statistically significant effects of lockdown measures and intermediate restrictions in reducing the number of consultations. The lockdown was associated with a reduction of 39.5% (p-value<0.001), while intermediate restrictions led to a 21.5% decline (p-value 0.017). These percentages increased to 41.8% (p-value<0.001) and 27.3% (p-value 0.001), respectively, when excluding COVID-19-related consultations from the analysis.

**Table 4:** Poisson regression model on number of consultations per working-day during lockdown and restrictions.

	Lockdown vs. no-restrictions		Intermediate restrictions vs. no-restrictions		Year 2020 vs. 2019	
	IRR (SE)	CI	IRR (SE)	CI	IRR (SE)	CI
**All consultations for outpatients**	**.521 (.100)**	**(.357; .759)**	**.716 (.093)**	**(.555; .923)**	**1.711 (.113)**	**(1.503; 1.946)**
Psychological consultations from GPs or other specialists	**.313 (.109)**	**(.159; .618)**	.721 (.146)	(.484; 1.073)	**1.878 (.182)**	**(1.553; 2.271)**
Psychological consultations from hospital wards	.803 (.163)	(.539; 1.196)	.853 (.206)	(.531; 1.370)	**1.580 (.141)**	**(1.326; 1.884)**
Psychiatric consultations from GPs or other specialists	.623 (.165)	(.370; 1.047)	.754 (.127)	(.542; 1.048)	**1.546 (.198)**	**(1.203; 1.987)**
Psychiatric consultations from hospital wards	.748 (.328)	(.317; 1.766)	**.400 (.109)**	**(.234; .682)**	**1.681 (.189)**	**(1.349; 2.095)**
**All consultations for inpatients**	.899 (.134)	(.670; 1.204)	0.990 (.139)	(.753; 1.303)	0.916 (.154)	(0.811; 1.034)
Consultations from surgical wards	1.192 (.422)	(.596; 2.385)	1.504 (.526)	(.758; 2.984)	**0.639 (.009)**	**(0.456; 0.896)**
Consultations from medical wards	.824 (.139)	(.593; 1.146)	.862 (.142)	(.624; 1.190)	1.034 (.073)	(0.900; 1.187)
Consultations from medical wards, excl. COVID-19-related	**.716 (.122)**	**(.513; .9996)**	**.542 (.089)**	**(.393; .747)**	1.017 (.074)	(0.882; 1.173)
All consultations for inpatients, excl. COVID-19-related	.813 (.121)	(.608; 1.088)	**.716 (.101)**	**(.544; .943)**	0.905 (.057)	(0.799; 1.023)
**All consultations**	**.605 (.074)**	**(.476; .768)**	**.785 (.079)**	**(.644; .958)**	**1.424 (.070)**	**(1.288; 1.565)**
**All consultations – excl. COVID-19-related**	**.582 (.074)**	**(.454; .745)**	**.727 (.070)**	**(.602; .878)**	**1.420 (.070)**	**(1.293; 1.568)**

IRR, Incidence Rate Ratio; SE, Standard Error; CI, Confidence Interval. Significant results are marked in bold.

The impact of these measures varied significantly (with a p-value of 0.020 when considering all consultations and a p-value of 0.026 when excluding those related to COVID-19) depending on the types of consultations. An instance of this can be seen in psychiatric consultations requested by GPs or other specialists, which exhibited a similar effect of the lockdown as of observed in overall consultations. However, this effect did not achieve statistical significance at the conventional threshold of 5% (p-value 0.074). The impact of restrictions and lockdown on psychological and psychiatric consultations for outpatient requested by hospital units, and on those for inpatients in medical wards was estimated to be comparatively lower, without statistical significance. There was a substantial reduction (68.7%; p-value 0.001) of psychological consultations requested by GPs or other specialists outside the hospital ascribed to the lockdown.

Regarding the impact of intermediate restrictions on psychological and psychiatric consultations requested by GPs or other specialists, psychological outpatient consultations requested by hospital units and inpatient consultations requested by medical 530 wards, results showed estimates comparable to those derived from the overall data, but no statistical signi!cance was found due to the smaller sample sizes. We estimated a 60% reduction (p-value 0.001) of psychiatric outpatient consultations within hospital units following the implementation of intermediate restrictions on contact.

For the number of consultations requested by surgical wards, the parameters displayed an opposite trend, indicating a positive estimated effect of both lockdown and restrictions. However, in both cases statistical significance was not reached.

When contacts were categorized into “all outpatient” vs “all inpatients” types, results were statistically significant for the former category only. In particular, both the lockdown (p-value 0.001, estimated reduction: 47.9%) and intermediate restrictions (p-value 0.010, estimated reduction: 28.4%) had a lowering effect on the number of outpatient consultations.

Furthermore, when COVID-19 related consultations were excluded from the analysis, we found a reduction of 28.4% due to the lockdown (p-value 0.0497), and of 45.8% due to intermediate restrictions (p-value<0.001) on medical ward contacts. We also found a decrease in the total number of inpatients consultations due to both COVID-19 containment measures. This reduction was specifically evident during intermediate restrictions (28.4%, p-value 0.017).

The number of contacts in 2020 would have been increased of 42.4% (p-value<0.001) compared to 2019, in case no restrictions were introduced. This is linked to outpatient consultations only (that would have shown, a 71.1% increase, p-value<0.001), while we estimate a reduction in consultations from surgical wards would have been observed (-36.1%; p-value 0.009).

## 4 Discussion

This is an observational retrospective study focused on the impact of the COVID-19 pandemic on CL psychiatric and psychological services conducted in a General Hospital in the Northeast Italy. The data clearly shows a significant increase in the number of requests for psychiatric and psychological consultations in 2020, concurrently with the COVID-19 pandemic, compared to the previous year.

This increase in demand is consistent with recent global reports on the mental health impact of the COVID-19 pandemic, which reported an increased request and need of mental health care (32–35). Despite the greater demand for psychiatric and psychological CL services, the lockdown was estimated to reduce consultations by 42.9%, with a smaller drop of 19.5% during the intermediate restriction phase.

This finding could potentially be explained by patients’ hesitance to access the hospital due to their pandemic-related fear (36). Despite the increased level of psychological distress experienced by the general population (37), distressed individuals might have tried to avoid or limit in-person meetings with healthcare providers (36). On the other hand, clinicians may have tried to manage hospital admissions cautiously, possibly to preserve hospital beds for potential increases in COVID-19 cases (2). This attitude could explain the decrease in CL psychiatry and psychology appointments. At this regard, in the Verona hospital during the pandemic phase, several wards were repurposed to admit COVID-19 patients. To minimize the infection spread, psychiatric and psychological consultations were often conducted via telephone or video calls. The Hospital Clinical Database was updated accordingly, to support the registration of teleconsultations. In light of the clinical demand observed during the pandemic, the implementation of telemedicine options - in particular in the field of psychiatric and psychological consultations – may be effective to meet the clinical needs of patients. Telemedicine has the potential to provide prompt treatment and preventive measures for patients, while also alleviating the strain on healthcare professionals (38).

Considering that, despite the significant effect of lockdown and restrictions in reducing the number of consultation requests, there was an increase in consultations in 2020, it is plausible that there was a rebound in demand once the number of COVID-19 cases decreased, and restrictions were reduced, to recover the previously lost consultations. Our results support this hypothesis, suggesting a potential increased demand for CL psychiatry and psychological services as the pandemic situation improved.

### 4.1 Limitations and strengths

Findings of the present study should be interpreted cautiously, as several methodological limitations are acknowledged. Firstly, our study faces a potential risk of information bias due to data collection conducted by various mental health professionals. This diversity in data collection methods has revealed gaps in sociodemographic data, which could potentially influence the study’s outcomes. Secondly, the registration of tele-consultation was implemented in the dataset only at the end of 2020. This delayed inclusion prevented us from separately analyzing the teleconsultation variable, which could have provided valuable insights for data interpretation. The inclusion of this information could have significantly contributed to our comprehension of the impact of tele-consultations on the observed trends. Thirdly, our dataset lacked critical information about reason for referral, individual clinical pathways and follow-ups. The absence of this data may potentially impact the comprehensive understanding of the factors affecting patients’ choices regarding seeking psychiatric or psychological consultations during and after the pandemic.

Lastly, our study was conducted within a single hospital, and it is essential to acknowledge the absence of certain departments, such as gynecology/obstetrics and pediatrics. Consequently, we must exercise caution when attempting to generalize the data across all specialties or other healthcare facilities or different regions in Italy. It is important to recognize that the Province of Verona experienced one of the most significant health impacts of the pandemic in Europe in terms of infections number (35, 39). Therefore, the unique circumstances of this heavily affected area may have influenced the observed trends, potentially limiting the applicability of our findings to other contexts.

In summary, the study compared the number of consultations in the years 2019 and 2020, and employed a robust “difference-in-differences” methodology. It explored multiple indicators and an appropriate regression model to reasonably estimate the effect of different level of pandemic-related restrictions on the number of consultations requests in 2020.

### 4.2 Implications

In terms of implications for further research, there is a need to expand the exploration to additional clinical and organizational factors that may influence the accessibility of psychiatric and psychological CL services. For example, evaluating specific individual factors such as socioeconomic status (40), diagnosis-related groups for each patient, service variables associated with healthcare system provisions and utilization, staff resources, or more general factors like the level of urbanization in relation to the use of CL services (41). A deeper understanding of these elements can offer valuable insights on the barriers and facilitators that impact the access to mental health support during periods of pandemic.

## 5 Conclusions

Our study identified an overall increase in the number of psychiatric and psychological consultations during 2020, despite a decreasing effect due to lockdown and intermediate restriction. To date, there are no observational studies investigating the long-term effects of the COVID-19 pandemic on psychiatric and psychological CL services. Further research and initiatives, that expand clinical and organizational factors impacting the accessibility of psychiatric and psychological CL services, are crucial for optimizing mental health care delivery during and beyond the pandemic (38). Implications from this study hold the potential to inform and shape policy and practice both at national and international level.

## Data Availability

The raw data supporting the conclusions of this article will be made available by the authors, without undue reservation.
